# Factory Life

**Published:** 1859-01

**Authors:** 


Factory Life.

It has rarely been our lot to read a series of more interesting letters than those in a pamphlet entitled *Experience of Factory Life*: by M. M.; reprinted from *The Waverley*, a Working Woman's Journal. The factory referred to is situated in a south eastern county; the factory work was performed, at the time the author of the pamphlet first visited the factory, by 937 women and girls, and about 76 men. The writer, hearing that the benevolent proprietors of the factory were looking out for some lady who would read and lecture to the girls, undertook herself this labour, and the little unassuming work chronicles her useful labours.

On first visiting her new residence, the writer found in the houses of the employed three great social sores all more or less connected with ignorance—viz., "careless and too early marriages; unsteady, idle conduct in the young women; and total disregard of all sanitary laws." To remedy these evils, there were established schools, an institute, a factory kitchen, a home, a nursery, an amusement society and a sick fund. The results of these institutions, in the course of a few short years, are such as to encourage every philanthropic heart, and we suggest without comment the perusal of these results by all our earnest fellow workers.

Amongst other works received by us this quarter we may notice Dr. Conway Evans's *Report on the Sanitary Condition of the Strand District*; a paper on *Marine Sanatoria*, by Dr. Morley Rooke, in which the author, while sustaining a controversy of a local kind, lays down some very sound rules as to the construction of these residences; a *Report of the Hygienic Congress at Copenhagen* in July 1858, an interesting and valuable document; and several numbers of a Danish sanitary periodical, containing a series of valuable papers, and edited by Dr. Hornemann, of Copenhagen.

